# Pharmacological Activation of NRF2 by Omaveloxolone Upregulates NRF2‐Target Proteins in SMA Type I Human Fibroblasts

**DOI:** 10.1096/fj.202601358R

**Published:** 2026-06-16

**Authors:** Sofia Vrettou, Sebastian Zetzsche, Brunhilde Wirth

**Affiliations:** ^1^ Institute of Human Genetics University Hospital of Cologne, University of Cologne Cologne Germany; ^2^ Center for Molecular Medicine Cologne University of Cologne Cologne Germany; ^3^ Center for Rare Diseases University Hospital of Cologne, University of Cologne Cologne Germany

**Keywords:** human fibroblasts, NRF2‐KEAP1 signaling, omaveloxolone, oxidative stress, redox homeostasis, spinal muscular atrophy

## Abstract

Spinal muscular atrophy (SMA) is caused by loss of SMN protein and is increasingly recognized as a multisystem disorder involving molecular pathology beyond motor neurons. Recently, we identified dysregulated NRF2‐KEAP1 signaling in SMA mice. Since NRF2 coordinates transcriptional programs that maintain cellular redox homeostasis and adaptive stress responses, we investigated whether NRF2 signaling is similarly altered in fibroblasts derived from individuals with SMA type I and whether it can be pharmacologically engaged. Compared with control fibroblasts, SMA fibroblasts displayed reduced basal expression of NRF2 target proteins, including NQO1 and xCT (SLC7A11), along with decreased levels of PGC1α. Omaveloxolone (OMAV), a pharmacological NRF2 activator approved for the treatment of Friedreich's ataxia, increased cell viability and upregulated NRF2 target proteins in both control and SMA fibroblasts. Notably, OMAV produced a modest increase in SMN protein abundance and PGC1α levels selectively in SMA cells. Together, these findings support diminished NRF2 pathway activity as a feature of SMA fibroblasts and demonstrate that OMAV activates NRF2 signaling in this human SMA cellular model, consistent with enhanced cytoprotective signaling. These results support further investigation of NRF2 activation, including OMAV, as a potential adjunctive strategy in SMA.

## Graphical abstract

SMA type I patient‐derived fibroblasts exhibit reduced basal NRF2 pathway output, reflected by decreased NQO1, xCT, and PGC1α. Pharmacological NRF2 activation with omaveloxolone (OMAV) increases cell viability and induces NRF2 target proteins in both control and SMA fibroblasts. OMAV also modestly increases SMN and PGC1α protein abundance selectively in SMA cells, supporting NRF2 pathway activation as a strategy to enhance cellular resilience in SMA.

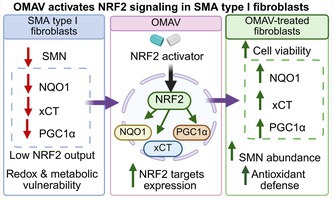

## 1 Introduction

Spinal muscular atrophy (SMA) is an autosomal recessive neuromuscular disorder caused by loss‐of‐function mutations in *SMN1*, resulting in insufficient levels of survival motor neuron (SMN) protein [1, 2]. Disease severity is modified by *SMN2* copy number, which partially compensates for *SMN1* loss through limited production of full‐length SMN [3]. SMN is ubiquitously present and participates in multiple fundamental cellular processes, including small nuclear ribonucleoprotein (snRNP) assembly and RNA metabolism [4]. Although motor neuron degeneration defines the clinical phenotype, accumulating evidence supports broader multisystem involvement, including metabolic dysfunction and redox imbalance in peripheral tissues [5, 6]. Consistent with this concept, our most recent multi‐organ analyses in SMA mouse models have revealed organ‐specific redox imbalance that is partially normalized following SMN restoration [7]. Moreover, we have shown that the NRF2‐KEAP1 pathway is dysregulated in this system [8].

The NRF2‐KEAP1 pathway is a central cytoprotective axis regulating detoxification enzymes, glutathione metabolism, and oxidative stress adaptation [9, 10]. Canonical NRF2 targets include NAD(P)H quinone dehydrogenase 1 (NQO1) and the cysteine‐glutamate antiporter SLC7A11 (xCT), which support redox buffering through quinone detoxification and cystine import for glutathione synthesis, respectively [11, 12]. Mitochondrial dysfunction and oxidative stress have been reported in SMA models, suggesting that redox imbalance contributes to cellular vulnerability beyond the neuromuscular compartment [13, 14, 15]. Prior work in human SMA muscle further reported reduced NRF1 and NRF2 expression alongside impaired mitochondrial biogenesis and antioxidant signaling [16]. In addition, antioxidant or NRF2‐associated compounds have been reported to increase SMN abundance in SMA‐related cellular models [14, 17, 18]. Patient‐derived fibroblasts provide a non‐neuronal cellular model to examine cell‐autonomous consequences of SMN deficiency, including altered redox, mitochondrial, and stress‐response pathways, independently of motor neuron‐specific degeneration [15, 19, 20]. This is relevant because SMA is increasingly recognized as a multisystem disorder involving peripheral tissues, where metabolic and redox abnormalities may contribute to disease complexity beyond the neuromuscular compartment [5, 6].

## 2 Results and Discussion

**FIGURE 1:**
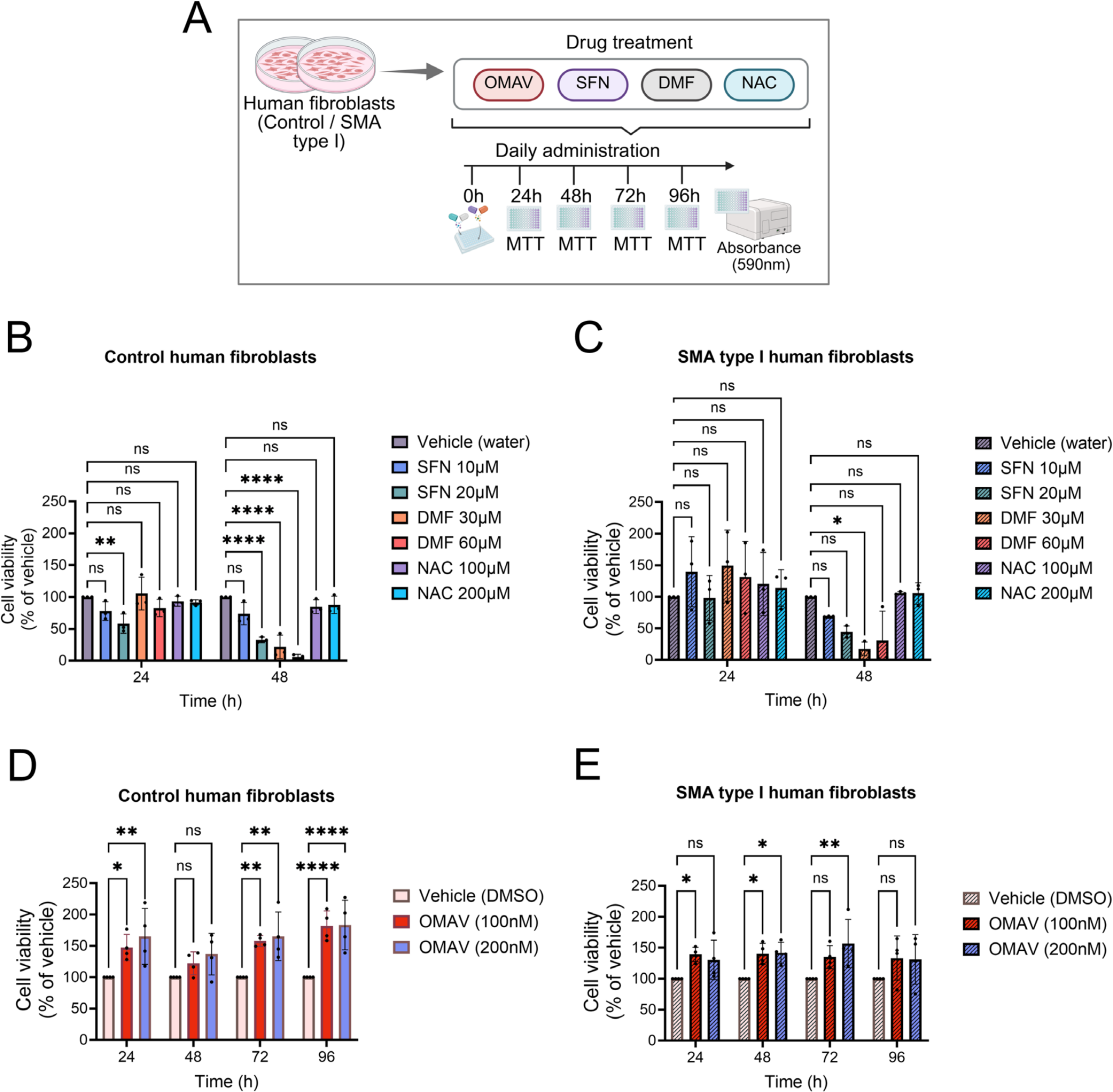
Pharmacological modulation of cell viability in control and SMA type I human fibroblasts. (A) Experimental design. Control and SMA type I human fibroblasts were treated daily with NRF2‐modulating compounds: sulforaphane (SFN), dimethyl fumarate (DMF), omaveloxolone (OMAV), or the antioxidant precursor N‐acetylcysteine (NAC), or the corresponding vehicles. Cell viability was assessed by MTT assay at the indicated time points (24, 48, 72, and 96 h); absorbance was measured at 590 nm. (B, C) Compound screen in control (B) and SMA type I (C) fibroblasts treated with SFN (10 or 20 μM), DMF (30 or 60 μM), NAC (100 or 200 μM). Viability was measured at 24 and 48 h. SFN, DMF, and NAC were prepared in water, and cell viability values were normalized to the corresponding vehicle controls and expressed as % cell viability. (D, E) OMAV time course in control (D) and SMA type I (E) fibroblasts treated with OMAV (100 or 200 nM) or vehicle (DMSO) with daily refreshment. Viability was measured at 24, 48, 72, and 96 h, normalized to DMSO vehicle control, and expressed as % cell viability. The final DMSO concentration in culture was ~0.001% (v/v). Data represent 3–4 independent fibroblast cell lines per genotype and are shown as mean ± SD. Statistical analysis was performed using one‐way ANOVA followed by Dunnett's multiple comparisons test within each timepoint. Statistical significance is indicated as: *p < 0.05; **p < 0.01; ****p < 0.0001; ns, not significant.

Endogenous antioxidant signaling in human SMA patient‐derived fibroblasts has not been fully defined. Here, we first assessed whether pharmacological modulation of NRF2 signaling influences cellular viability. Control (*SMN1*: 2 copies; *SMN2*: 1–2 copies) and SMA type I (*SMN1*: 0 copies; *SMN2*: 2 copies) fibroblasts were treated with NRF2‐modulating compounds including sulforaphane (SFN), dimethyl fumarate (DMF), and the antioxidant precursor N‐acetylcysteine (NAC). Cells were exposed to two concentrations of each compound and cell viability was monitored using the MTT assay following daily compound administration (Figure 1A).

In control fibroblasts, SFN reduced viability in a dose‐ and time‐dependent manner, with 20 μM SFN decreasing viability to 58.1% at 24 h and 32.7% at 48 h. DMF showed a stronger time‐dependent effect, reducing viability to 21.7% at 30 μM and 5.7% at 60 μM after 48 h, whereas NAC remained close to vehicle‐treated levels (Figure 1B). A similar pattern was observed in SMA type I fibroblasts at 48 h, where 20 μM SFN reduced viability to 44.3%, and DMF reduced viability to 17.6% at 30 μM and 30.8% at 60 μM, whereas NAC remained near vehicle‐treated levels (Figure 1C).

We next examined the impact of omaveloxolone (OMAV), a potent pharmacological NRF2 activator approved for the treatment of Friedreich's ataxia [21], on SMA cells. OMAV was well tolerated and increased viability in both genotypes, although with different temporal profiles. In control fibroblasts, OMAV increased viability at 24, 72, and 96 h, reaching 181.9% with 100 nM and 183.2% with 200 nM at 96 h (Figure 1D). In SMA type I fibroblasts, OMAV increased viability earlier, reaching 140.2% with 100 nM and 142.0% with 200 nM at 48 h, with 200 nM OMAV reaching 156.8% at 72 h (Figure 1E).

The above screening results were not intended as a head‐to‐head efficacy comparison but reflect compound‐specific differences in pharmacology and tolerability under the conditions tested.

**FIGURE 2:**
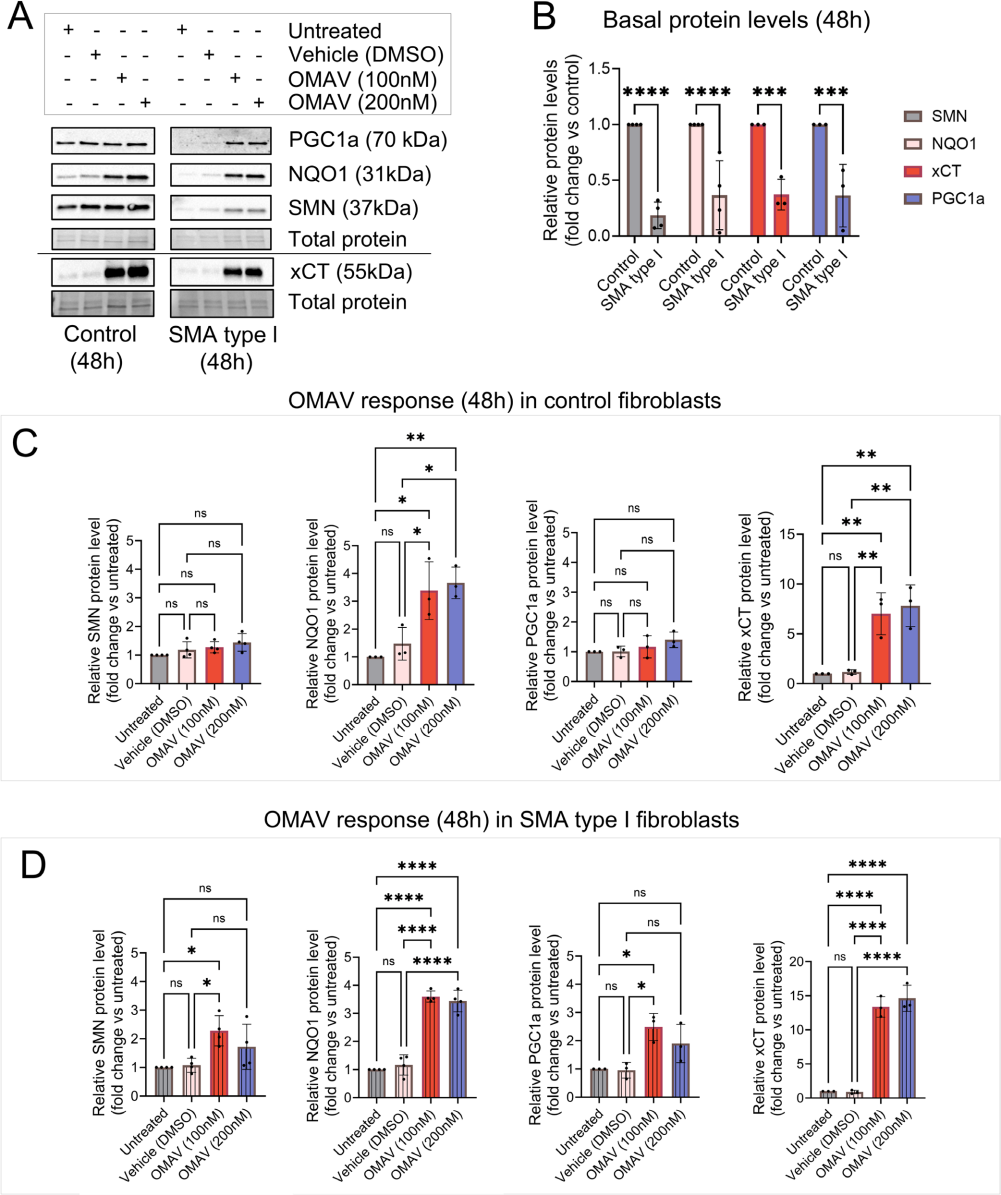
Reduced basal NRF2 target proteins in SMA type I fibroblasts and induction by omaveloxolone. (A) Representative Western blots from control and SMA type I fibroblasts treated for 48 h with omaveloxolone (OMAV; 100 or 200 nM), vehicle (DMSO), or left untreated. Membranes were probed with antibodies against SMN, NQO1, PGC1α, and xCT. Total protein staining was used as the loading control for normalization. (B) Basal protein levels (untreated) in control versus SMA type I fibroblasts, normalized to total protein and expressed relative to control (set to 1) for each target. Statistical analysis was performed using two‐way ANOVA with Šídák's multiple‐comparisons test comparing control vs. SMA for each protein target. (C, D) OMAV response in control (C) and SMA type I (D) fibroblasts treated with OMAV (100 or 200 nM). Protein abundance was normalized to total protein and expressed as fold change relative to the untreated condition (set to 1), since vehicle (DMSO) produced a mild increase in SMN in a subset of cell lines. Statistical comparisons are shown for untreated vs. all conditions and for vehicle (DMSO) vs. all conditions, as indicated on the graphs. Panels C, D were analyzed by one‐way ANOVA followed by Šídák's multiple‐comparisons test. Dots represent independent fibroblast cell lines and are presented as mean ± SD. Three control and three SMA type I cell lines were analyzed; one line pair was repeated in an independent experiment, yielding *n* = 4 for SMN and NQO1 and *n* = 3 for xCT and PGC1α. Statistical significance is indicated as: *p < 0.05; **p < 0.01; ***p < 0.001; ****p < 0.0001; ns, not significant.

Because OMAV‐associated viability changes were already detectable at 48 h, and preliminary time‐course Western blot analyses indicated that NRF2‐associated protein responses reached a plateau by 48 h, we selected this time point for protein analysis. Cells were therefore treated with OMAV every 24 h for 48 h prior to Western blot analysis (Figure 2). Because DMSO can influence cellular gene/protein expression and fibroblast behavior in vitro [22, 23], OMAV responses were expressed relative to untreated cells when DMSO caused a mild SMN increase in a subset of lines, while vehicle controls are shown for transparency.

Western blot analysis revealed reduced basal levels of NRF2 target proteins in SMA fibroblasts compared with controls (Figure 2A,B). Relative to control levels, SMA fibroblasts showed reduced SMN (0.19‐fold, *p* < 0.0001), NQO1 (0.37‐fold, *p* < 0.0001), xCT (0.37‐fold, *p* = 0.0005), and peroxisome proliferator‐activated receptor gamma coactivator‐1 alpha (PGC1α) (0.36‐fold, *p* = 0.0004) (Figure 2A,B). PGC1α is a key regulator of mitochondrial metabolism and biogenesis [24]. Although PGC1α is not a canonical NRF2 target, functional crosstalk between NRF2 signaling and PGC1α‐dependent metabolic programs has been reported [25]. The reduction of PGC1α observed here is therefore consistent with growing evidence that SMN deficiency is associated with metabolic and mitochondrial perturbations in patient‐derived fibroblasts, including recent reports of mitochondrial dysfunction even in fibroblasts from SMA carriers [20].

In control fibroblasts, OMAV increased NQO1 and xCT, with 100 nM OMAV inducing NQO1 to 3.39 ± 1.04‐fold and xCT to 7.02 ± 2.10‐fold of untreated levels, while PGC1α and SMN remained unchanged (Figure 2A,C). In SMA fibroblasts, 100 nM OMAV induced NQO1, xCT, and PGC1α to 3.60 ± 0.20, 13.38 ± 1.51, and 2.49 ± 0.48‐fold of untreated levels, respectively, and significantly increased SMN to 2.28 ± 0.53‐fold (*p* = 0.0153; *n* = 4 for SMN and NQO1; *n* = 3 for xCT and PGC1α) (Figure 2A,D). These findings indicate that OMAV activates NRF2‐associated target expression in both control and SMA fibroblasts, while selectively increasing PGC1α and SMN in SMA cells. *SMN2* copy‐number differences may contribute to interline variability, although stratified analysis was not feasible given the small number of independent fibroblast lines.

The modest SMN increase observed in SMA fibroblasts should be considered hypothesis‐generating. One possible explanation is that OMAV‐induced NRF2 activation improves the intracellular stress environment through increased expression of antioxidant and cytoprotective target genes [10], thereby reducing redox‐associated proteostatic stress and favoring SMN protein stability. This interpretation is consistent with evidence that cellular stress and redox imbalance can influence SMN stability, turnover, complex assembly, and transcript processing [14, 26, 27, 28]. Because oxidative stress has also been linked to aberrant *SMN2* splicing [28], improved redox homeostasis could, in principle, indirectly affect *SMN2*‐derived SMN production. A more direct connection between NRF2 signaling and SMN biology was recently suggested by work showing that NRF2 can regulate *SMN1* transcription through antioxidant response elements and physically associate with SMN‐containing nuclear complexes [29]. Although these observations were made outside the SMA context, and the SMA fibroblasts used here lack *SMN1*, they provide a framework through which NRF2 activation could influence SMN‐associated ribonucleoprotein biology. Thus, OMAV may increase SMN levels through improved redox and proteostatic homeostasis, altered *SMN2*‐dependent expression or splicing, translation, protein stability, or a combination of these mechanisms.

From a translational perspective, OMAV is notable because it is clinically approved for Friedreich's ataxia [21], a neurodegenerative and multisystem disorder involving impaired mitochondrial homeostasis and oxidative stress [30]. OMAV may therefore warrant future evaluation as a potential adjunct strategy in SMA. In combination with approved therapies that restore *SMN2* splicing or increase SMN expression [31], OMAV may complement SMN‐targeted approaches by mitigating cellular stress and redox imbalance, thereby addressing pathogenic mechanisms that are not directly corrected by SMN restoration alone [3, 15].

Collectively, our findings demonstrate that:

1. SMA type I human fibroblasts exhibit reduced basal expression of NRF2 target proteins, including NQO1 and xCT, together with decreased PGC1α.
2. OMAV increases expression of NRF2 target proteins in both control and SMA fibroblasts.
3. OMAV modestly increases SMN protein abundance selectively in SMA fibroblasts.

These results identify reduced NRF2 pathway output as a feature of SMN‐deficient fibroblasts and support further investigation of the mechanistic relationship between NRF2‐dependent stress‐response signaling and SMN regulation.

## Author Contributions

**Sofia Vrettou:** designed the work, performed all the experiments, analyzed the results, and wrote the manuscript. **Sebastian Zetzsche:** involved in cell culture experiments. **Brunhilde Wirth:** involved in conceptualization, reviewed and edited the manuscript, supervised data acquisition, and provided funding.

## Funding

This work was supported by the European Union's Horizon 2020 Marie Skłodowska‐Curie Program (project 956185; SMABEYOND) to B.W. and the Center for Molecular Medicine Cologne (project C18) to B.W.

## Ethics Statement

Informed written consent was obtained from individuals with SMA and controls according to the Declaration of Helsinki, and the study was approved by the ethics committee of the University Hospital of Cologne under the approval numbers 04‐138 and 13‐022.

## Conflicts of Interest

The authors declare no conflicts of interest.

## Supporting information


**Table S1:** Compounds, vehicles, and treatment conditions.
**Table S2:** Antibodies used for Western blotting.

## Data Availability

All data supporting the findings of this study have been deposited in Zenodo and are publicly available at: https://doi.org/10.5281/zenodo.19068620.

## References

[1] T. Prior , M. Leach , and E. Finanger , “Spinal Muscular Atrophy,” in GeneReviews (University of Washington, Seattle, 2000), https://www.ncbi.nlm.nih.gov/books/NBK1352/. 20301526

[2] S. Lefebvre , L. Bürglen , S. Reboullet , et al., “Identification and Characterization of a Spinal Muscular Atrophy‐Determining Gene,” Cell 80 (1995): 155–165, 10.1016/0092-8674(95)90460-3. 7813012

[3] B. Wirth , “Spinal Muscular Atrophy: In the Challenge Lies a Solution,” Trends in Neurosciences 44 (2021): 306–322, 10.1016/j.tins.2020.11.009. 33423791

[4] R. N. Singh , M. D. Howell , E. W. Ottesen , and N. N. Singh , “Diverse Role of Survival Motor Neuron Protein,” Biochimica et Biophysica Acta, Gene Regulatory Mechanisms 1860 (2017): 299–315, 10.1016/j.bbagrm.2016.12.008. 28095296 PMC5325804

[5] C. J. J. Yeo and B. T. Darras , “Overturning the Paradigm of Spinal Muscular Atrophy as Just a Motor Neuron Disease,” Pediatric Neurology 109 (2020): 12–19, 10.1016/j.pediatrneurol.2020.01.003. 32409122

[6] M. Shababi , J. Habibi , H. T. Yang , S. M. Vale , W. A. Sewell , and C. L. Lorson , “Cardiac Defects Contribute to the Pathology of Spinal Muscular Atrophy Models,” Human Molecular Genetics 19 (2010): 4059–4071, 10.1093/hmg/ddq329. 20696672

[7] S. Vrettou and B. Wirth , “Organ‐Specific Redox Imbalances in Spinal Muscular Atrophy Mice Are Partially Rescued by SMN Antisense Oligonucleotides,” FEBS Letters (2026), 10.1002/1873-3468.70303. Epub ahead of print. PMC13502620 41684306

[8] S. Vrettou , S. Müller , and B. Wirth , “SMN Deficiency Disrupts Hepatic Mitochondrial Iron Homeostasis and NRF2‐Dependent Redox Control in Spinal Muscular Atrophy,” bioRxiv (Preprint). 2026.01.08.698518 (2026), 10.64898/2026.01.08.698518.

[9] T. Nguyen , P. Nioi , and C. B. Pickett , “The Nrf2‐Antioxidant Response Element Signaling Pathway and Its Activation by Oxidative Stress,” Journal of Biological Chemistry 284 (2009): 13291–13295, 10.1074/jbc.R900010200. 19182219 PMC2679427

[10] J. D. Hayes and A. T. Dinkova‐Kostova , “The Nrf2 Regulatory Network Provides an Interface Between Redox and Intermediary Metabolism,” Trends in Biochemical Sciences 39 (2014): 199–218, 10.1016/j.tibs.2014.02.002. 24647116

[11] M. Dodson , M. R. de la Vega , A. B. Cholanians , C. J. Schmidlin , E. Chapman , and D. D. Zhang , “Modulating NRF2 in Disease: Timing Is Everything,” Annual Review of Pharmacology and Toxicology 59 (2019): 555–575, 10.1146/annurev-pharmtox-010818-021856. PMC6538038 30256716

[12] L. Baird and M. Yamamoto , “The Molecular Mechanisms Regulating the KEAP1‐NRF2 Pathway,” Molecular and Cellular Biology 40 (2020): 20, 10.1128/mcb.00099-20. PMC7296212 32284348

[13] E. Zilio , V. Piano , and B. Wirth , “Mitochondrial Dysfunction in Spinal Muscular Atrophy,” International Journal of Molecular Sciences 23 (2022): 10878, 10.3390/ijms231810878. 36142791 PMC9503857

[14] M. P. Thelen , B. Wirth , and M. J. Kye , “Mitochondrial Defects in the Respiratory Complex I Contribute to Impaired Translational Initiation via ROS and Energy Homeostasis in SMA Motor Neurons,” Acta Neuropathologica Communications 8 (2020): 223, 10.1186/s40478-020-01101-6. 33353564 PMC7754598

[15] R. James , H. Chaytow , L. M. Ledahawsky , and T. H. Gillingwater , “Revisiting the Role of Mitochondria in Spinal Muscular Atrophy,” Cellular and Molecular Life Sciences 78 (2021): 4785–4804, 10.1007/s00018-021-03819-5. 33821292 PMC8195803

[16] M. Ripolone , D. Ronchi , R. Violano , et al., “Impaired Muscle Mitochondrial Biogenesis and Myogenesis in Spinal Muscular Atrophy,” JAMA Neurology 72 (2015): 666–675, 10.1001/jamaneurol.2015.0178. 25844556 PMC4944827

[17] Y. Y. Hsu , C. S. Chen , S. N. Wu , Y. J. Jong , and Y. C. Lo , “Berberine Activates Nrf2 Nuclear Translocation and Protects Against Oxidative Damage via a Phosphatidylinositol 3‐Kinase/Akt‐Dependent Mechanism in NSC34 Motor Neuron‐Like Cells,” European Journal of Pharmaceutical Sciences 46 (2012): 415–425, 10.1016/j.ejps.2012.03.004. 22469516

[18] R. Adami , M. Pezzotta , F. Cadile , et al., “Physiological Features of the Neural Stem Cells Obtained From an Animal Model of Spinal Muscular Atrophy and Their Response to Antioxidant Curcumin,” International Journal of Molecular Sciences 25, no. 15 (2024): 8364. 39125934 10.3390/ijms25158364 PMC11313061

[19] A. S. Köstel , G. Bora‐Tatar , and H. Erdem‐Yurter , “Spinal Muscular Atrophy: An Oxidative Stress Response Counteracted With Curcumin,” Biomedicine & Aging Pathology 2 (2012): 61–66, 10.1016/j.biomag.2012.03.007.

[20] R. James , K. M. E. Faller , E. J. N. Groen , B. Wirth , and T. H. Gillingwater , “Altered Mitochondrial Function in Fibroblast Cell Lines Derived From Disease Carriers of Spinal Muscular Atrophy,” Communications Medicine 4 (2024): 86, 10.1038/s43856-024-00515-w. 38750213 PMC11096342

[21] A. Lee , “Omaveloxolone: First Approval,” Drugs 83 (2023): 725–729, 10.1007/s40265-023-01874-9. 37155124

[22] M. Verheijen , M. Lienhard , Y. Schrooders , et al., “DMSO Induces Drastic Changes in Human Cellular Processes and Epigenetic Landscape In Vitro,” Scientific Reports 9 (2019): 4641, 10.1038/s41598-019-40660-0. 30874586 PMC6420634

[23] M. Singh , “Effect of Dimethyl Sulfoxide on In Vitro Proliferation of Skin Fibroblast Cells,” Journal of Biotech Research 8 (2017): 78–82.

[24] R. C. Scarpulla , “Metabolic Control of Mitochondrial Biogenesis Through the PGC‐1 Family Regulatory Network,” Biochimica et Biophysica Acta 1813 (2011): 1269–1278, 10.1016/j.bbamcr.2010.09.019. 20933024 PMC3035754

[25] A. P. Gureev , E. A. Shaforostova , and V. N. Popov , “Regulation of Mitochondrial Biogenesis as a Way for Active Longevity: Interaction Between the Nrf2 and PGC‐1α Signaling Pathways,” Frontiers in Genetics 10 (2019): 435, 10.3389/fgene.2019.00435. 31139208 PMC6527603

[26] L. Wan , E. Ottinger , S. Cho , and G. Dreyfuss , “Inactivation of the SMN Complex by Oxidative Stress,” Molecular Cell 31 (2008): 244–254, 10.1016/j.molcel.2008.06.004. 18657506 PMC2867055

[27] N. N. Singh , J. Seo , S. J. Rahn , and R. N. Singh , “A Multi‐Exon‐Skipping Detection Assay Reveals Surprising Diversity of Splice Isoforms of Spinal Muscular Atrophy Genes,” PLoS One 7 (2012): e49595, 10.1371/journal.pone.0049595. 23185376 PMC3501452

[28] J. Seo , N. N. Singh , E. W. Ottesen , S. Sivanesan , M. Shishimorova , and R. N. Singh , “Oxidative Stress Triggers Body‐Wide Skipping of Multiple Exons of the Spinal Muscular Atrophy Gene,” PLoS One 11 (2016): e0154390, 10.1371/journal.pone.0154390. 27111068 PMC4844106

[29] Q. Cui , W. Wang , A. Namani , et al., “NRF2 Has a Splicing Regulatory Function Involving the Survival of Motor Neuron (SMN) in Non‐Small Cell Lung Cancer,” Oncogene 42 (2023): 2751–2763, 10.1038/s41388-023-02799-z. 37573407

[30] R. Abeti , A. Baccaro , N. Esteras , and P. Giunti , “Novel Nrf2‐Inducer Prevents Mitochondrial Defects and Oxidative Stress in Friedreich's Ataxia Models,” Frontiers in Cellular Neuroscience 12 (2018): 188, 10.3389/fncel.2018.00188. 30065630 PMC6056642

[31] E. Mercuri , C. J. Sumner , F. Muntoni , B. T. Darras , and R. S. Finkel , “Spinal Muscular Atrophy,” Nature Reviews Disease Primers 8 (2022): 52, 10.1038/s41572-022-00380-8. 35927425

