# Supplementary material for: Pharmacological Activation of NRF2 by Omaveloxolone Upregulates NRF2‐Target Proteins in SMA Type I Human Fibroblasts
Source: FASEB J. 2026 Jun 16;40(12):e72064. doi: 10.1096/fj.202601358R (PMC13271045; doi:10.1096/fj.202601358R)
Supplement: Supplementary file 1 — Table S1: Compounds, vehicles, and treatment conditions. Table S2: Antibodies used for Western blotting. [file FSB2-40-e72064-s001.docx]

**Supplementary Methods**

*Pharmacological Activation of NRF2 by Omaveloxolone Upregulates NRF2-Target Proteins in SMA Type I Human Fibroblasts*

Sofia Vrettou^1,2^, Sebastian Zetzsche^2^, Brunhilde Wirth^1,2,3^

^1^Institute of Human Genetics, University Hospital of Cologne, University of Cologne, 50931 Cologne, Germany.

^2^Center for Molecular Medicine Cologne, University of Cologne, 50931 Cologne, Germany.

^3^Center for Rare Diseases, University Hospital of Cologne, University of Cologne, 50931 Cologne, Germany.

Corresponding author: Sofia Vrettou, [sofia.vrettou@uk-koeln.de](mailto:sofia.vrettou@uk-koeln.de)

**Cell lines and characterization**

**Human fibroblast lines**

Human primary fibroblast lines derived from skin biopsies of control individuals and individuals with SMA type I were used. Informed written consent was obtained from all individuals according to the Declaration of Helsinki, and the study was approved by the ethics committee of the University Hospital of Cologne under the approval numbers 04-138 and 13-022.

Fibroblast lines were selected based on their *SMN1* and *SMN2* copy-number status. Control fibroblast lines carried two *SMN1* copies and one (N=1) or two *SMN2* copies (N=3). SMA type I fibroblast lines carried zero *SMN1* copies and two *SMN2* copies (N=4).

***SMN1* and *SMN2* copy-number assessment**

*SMN1* and *SMN2* copy number was assessed by multiplex ligation-dependent probe amplification (MLPA) using the SALSA MLPA system according to the manufacturer’s instructions (MRC Holland; SALSA MLPA Reagent Kit EK5-FAM, cat. no. EK5-FAM).

**Culture conditions**

Primary fibroblasts were cultured in Dulbecco’s Modified Eagle Medium (D-MEM; Gibco, cat. no. 41966-029) containing 4.5 g/L glucose, L-glutamine, and pyruvate. The medium was supplemented with 10–15% fetal calf serum (FCS), 1% penicillin/streptomycin (Gibco cat. no. 15140-122), and amphotericin B (PromoCell #C42040). Cells were maintained at 37°C in a humidified incubator with 5% CO₂ in 75 cm² tissue culture flasks containing 12 mL complete medium.

**Routine passaging**

Fibroblasts were passaged when they reached approximately 70-80% confluency. Depending on proliferation rate, cells were split at ratios ranging from 1:2 to 1:4. For passaging, culture medium was removed and cells were washed with 5 mL PBS-Dulbecco without Mg²⁺/Ca²⁺ (Biochrom, cat. no. L1825). Cells were detached using 2.5 mL trypsin (Sigma, cat. no. T3924) for approximately 5 min at 37°C. Trypsinization was stopped by adding 5 mL complete medium. Cells were gently resuspended and reseeded into new flasks with fresh medium to a final volume of 12 mL. Cells were expanded and used at the lowest feasible passage number to minimize passage-associated changes in fibroblast growth.

**Cell seeding for experimental assays**

For MTT viability assays, fibroblasts were seeded in 96-well plates at densities adjusted according to the planned treatment duration. For short-term assays ending at 24-48 h, cells were seeded at approximately 5,000 cells/well in 100µL complete medium. For longer time-course experiments extending to 72-96 h, cells were seeded at approximately 3,000 cells/well in 100µL complete medium to prevent overconfluence during prolonged culture. Cells were allowed to adhere for at least 24 h before treatment initiation.

For Western blot experiments, fibroblasts were seeded in 24-well plates at 10,000 cells/well in 500µL complete medium and treated at comparable confluency across control and SMA lines for 48h. Cells were allowed to adhere for at least 24 h before treatment initiation.

**Compound preparation and dosing schedule**

Sulforaphane (SFN), dimethyl fumarate (DMF), N-acetylcysteine (NAC), and omaveloxolone (OMAV) (Supplementary Table 1) were prepared as stock solutions using the solvents indicated below. For the initial viability screen, fibroblasts were treated daily with SFN (10 or 20 µM), DMF (30 or 60 µM), or NAC (100 or 200 µM). For OMAV experiments, cells were treated with OMAV at 100 nM or 200 nM, with daily compound refreshment. Vehicle controls were included in parallel. OMAV was prepared in DMSO, and the final DMSO concentration in culture medium was approximately 0.001% (v/v). For Western blot experiments, cells were treated for 48 h because the viability assay showed a detectable OMAV-associated response at this time point, allowing protein-level changes to be assessed within the early response window before longer-term culture effects.

**MTT viability assay**

Cell viability was assessed using the MTT assay kit from Abcam (cat. no. ab211091) according to the manufacturer’s instructions, with minor adaptations. Fibroblasts were seeded in transparent 96-well plates (Greiner) and allowed to adhere for 24 h before treatment initiation. SFN, DMF, and NAC were prepared in water, whereas OMAV was prepared in DMSO.

Following drug treatment for the indicated durations, culture medium was aspirated and replaced with 50 µL serum-free medium and 50 µL MTT reagent per well. Both the MTT reagent and MTT solvent were pre-warmed to 37°C before use. Plates were incubated with MTT reagent for 3 h at 37°C. Subsequently, 150 µL MTT solvent was added to each well, and plates were protected from light with aluminum foil and gently shaken at room temperature to solubilize the formazan product. Absorbance was measured at 590 nm using a Tecan Sapphire plate reader.

Blank wells containing serum-free medium, MTT reagent, and MTT solvent without cells were included as negative controls, and the corresponding background absorbance was subtracted from all experimental wells. For SFN, DMF, and NAC experiments, values were normalized to the corresponding water-treated vehicle controls and expressed as percentage cell viability. For OMAV experiments, values were normalized to DMSO-treated vehicle controls and expressed as percentage cell viability.

**Western blot sample preparation and electrophoresis**

Fibroblasts were lysed in ice-cold RIPA buffer (Sigma, cat. no. R0278), supplemented with protease and phosphatase inhibitor cocktails (Thermo Scientific Pierce, cat. no. A32961). Lysates were rocked for 30 min at 4°C and then centrifuged for 10 min at maximum speed at 4°C. Protein concentrations were determined by Bradford assay using Bradford reagent from AppliChem (cat. no. A6932,0500).

For SDS-PAGE, 30-40 µg protein was mixed with 1x Laemmli buffer containing 5% β-mercaptoethanol, heated at 95°C for ≤3 min, and resolved on 4-20% SDS-PAGE gradient gels (Bio-Rad, cat. no. 4561094). Proteins were transferred to PVDF or nitrocellulose membranes using the Bio-Rad Trans-Blot® Turbo™ Transfer System in semi-dry mode for 12 min.

Following transfer, membranes were stained for total protein using Pierce™ Reversible Protein Stain Kit for PVDF Membranes (Thermo Scientific, cat. no. 24585) or Pierce™ Reversible Protein Stain Kit for Nitrocellulose Membranes (Thermo Scientific, cat. no. 24580), according to the membrane type and manufacturer’s instructions. Total protein staining was used for loading normalization.

Membranes were blocked with 5% (w/v) non-fat milk in TBST containing 20 mM Tris-HCl pH 7.6, 150 mM NaCl, and 0.1% Tween-20 for ≥1 h at room temperature. Primary antibodies (Supplementary Table 2) were incubated either for 2 h at room temperature or overnight at 4°C in TBST containing 5% milk. After washing three times for 5 min each in TBST, membranes were incubated with HRP-conjugated anti-rabbit secondary antibody (Cell Signaling Technology, cat. no. 7074; 1:3000) or HRP-conjugated anti-mouse secondary antibody (Dianova, cat. no. 115-035-003; 1:5000) for at least 1 h at room temperature. Membranes were then washed again three times for 5 min each in TBST. Chemiluminescent signal detection was performed using SuperSignal™ West Pico PLUS chemiluminescence reagent (Pierce, cat. no. 34580) according to standard protocols.

**Image acquisition, quantification, and normalization**

Western blot images were acquired using a Bio-Rad imaging system and analyzed by densitometry using ImageJ/Fiji. Chemiluminescent signals were acquired under exposure conditions selected to avoid saturation and to remain within the detectable linear range. Target protein band intensities were quantified in ImageJ/Fiji after background subtraction. For total protein normalization, the corresponding total protein lane/column signal was quantified using volume intensity measurements rather than individual band intensity, with background subtraction applied to the total protein signal. For each sample, the target protein band intensity was divided by the corresponding total protein volume intensity from the same lane. The resulting normalized protein abundance values were then expressed relative to the indicated reference condition, either vehicle-treated or untreated cells, depending on the comparison described in the figure legends.

**Statistical analysis**

Statistical analyses and graph generation were performed using GraphPad Prism 10. For viability assays (MTT), one-way ANOVA followed by Dunnett's multiple-comparisons test was used to compare treatment groups with the corresponding vehicle control within each time point. Dunnett's test was selected because the viability experiments were designed to compare multiple compound doses against a single vehicle-treated reference condition. For Western blot comparisons, Šídák's multiple-comparisons test was used following ANOVA because defined pairwise comparisons were planned across genotype or treatment conditions while controlling for multiple testing. Data are presented as mean ± SD, with individual dots representing independent fibroblast cell lines or independent line-level measurements as indicated in the figure legends.

**Supplementary Table 1. Compounds, vehicles, and treatment conditions**

| Compound | Supplier | Catalog number | Stock solvent | Working concentration(s) | Final vehicle concentration | Treatment schedule |
| --- | --- | --- | --- | --- | --- | --- |
| Omaveloxolone/ RTA 408 (OMAV) | MedChemExpress | HY-12212 | DMSO | 100 nM; 200 nM | ~0.001% v/v DMSO | Daily treatment; viability readout at 24, 48, 72, and 96 h; Western blot at 48 h |
| Dimethyl fumarate (DMF) | MedChemExpress | HY-17363 | Water | 30 µM; 60 µM | *Equivalent volume of sterile water | Daily treatment; viability readout at 24 h and 48 h |
| Sulforaphane   (SFN) | MedChemExpress | HY-13755B | Water | 10 µM; 20 µM | *Equivalent volume of sterile water | Daily treatment; viability readout at 24 h and 48 h |
| N-Acetyl-L-cysteine (NAC) | Sigma | A9165 | Water | 100 µM; 200 µM | *Equivalent volume of sterile water | Daily treatment; viability readout at 24 h and 48 h |

*Equivalent volume of sterile water, matching the highest treatment-volume condition.

**Supplementary Table 2. Antibodies used for Western blotting**

| **Target** | **Host/clonality** | **Supplier** | **Catalog number** | **Dilution** | **Incubation** |
| --- | --- | --- | --- | --- | --- |
| SMN | Mouse monoclonal IgG1, clone 8/SMN | BD Transduction Laboratories | 610647 | 1 in 1000 | 2h RT |
| NQO1 | Rabbit monoclonal | Cell Signaling Technology | 62262 | 1 in 800 | Overnight at 4^o^C |
| xCT/SLC7A11 | Rabbit monoclonal | Cell Signaling Technology | 12691 | 1 in 500 | Overnight at 4^o^C |
| PGC1α | Mouse monoclonal IgG1 | Proteintech | 66369-1-Ig | 1 in 500 | Overnight at 4^o^C |
| Anti-rabbit IgG, HRP-linked | Goat | Cell Signaling Technology | 7074 | 1:3000 | ≥1h RT |
| Anti-Mouse IgG (H+L), HRP-linked | Goat polyclonal | Dianova | 115-035-003 | 1:5000 | ≥1h RT |
